# Clinically Relevant Risk of Hepatitis B Virus Infection and Reactivation in People With HIV After Withdrawal of Anti-HBV–Active Antiretroviral Therapy in Japan

**DOI:** 10.1093/ofid/ofag533

**Published:** 2026-08-20

**Authors:** Eisuke Adachi, Aiko Okazaki, Yoshiaki Kanno, Michiko Koga, Hiroshi Yotsuyanagi

**Affiliations:** Department of Infectious Diseases and Applied Immunology, IMSUT Hospital of the Institute of Medical Science, the University of Tokyo, Tokyo, Japan; Department of Infectious Diseases and Applied Immunology, IMSUT Hospital of the Institute of Medical Science, the University of Tokyo, Tokyo, Japan; Department of Infectious Diseases and Applied Immunology, IMSUT Hospital of the Institute of Medical Science, the University of Tokyo, Tokyo, Japan; Department of Infectious Diseases and Applied Immunology, IMSUT Hospital of the Institute of Medical Science, the University of Tokyo, Tokyo, Japan; Department of Infectious Diseases and Applied Immunology, IMSUT Hospital of the Institute of Medical Science, the University of Tokyo, Tokyo, Japan

**Keywords:** ART without anti-HBV activity, HBV, HBV reactivation, HIV, men who have sex with men

## Abstract

**Background:**

In people with HIV-1 (PWH), antiretroviral therapy (ART) containing anti-HBV drugs provides protection against hepatitis B virus (HBV) infection. However, the risk of HBV infection or reactivation after switching to ART without anti-HBV activity remains unclear, particularly in settings with recent implementation of universal HBV vaccination.

**Methods:**

We conducted a single-center retrospective cohort study of PWH who received ART regimens without anti-HBV activity between January 2005 and March 2026. HBV serological status at baseline was classified, and participants were followed during periods on ART without tenofovir, lamivudine, or emtricitabine. Incident HBV infection and HBV reactivation were defined based on serological and virological criteria.

**Results:**

A total of 166 PWH were included, with 716 person-years (PY) of follow-up. Five HBV-related events were observed. Two occurred in HBV-naïve individuals and were consistent with incident HBV infection based on seroconversion to anti-HBc and anti-HBs positivity. Two cases with isolated anti-HBc positivity were classified as suspected HBV reactivation based on marked increases in anti-HBs titers. The remaining case with isolated anti-HBc positivity was confirmed HBV reactivation. The overall incidence rate was .70 per 100 PY, increasing to 1.28 per 100 PY in anti-HBs–negative individuals. Kaplan–Meier analysis showed lower event-free survival in anti-HBs–negative individuals compared with those with anti-HBs positivity. (log-rank *P* = .0084).

**Conclusions:**

In PWH receiving ART without anti-HBV activity, the risk of HBV infection and reactivation becomes apparent, particularly among those without anti-HBs, especially those with isolated anti-HBc, in settings with limited impact of universal HBV vaccination.

## Teaser

This study evaluates HBV reactivation and incident infection risk among people with HIV after switching to ART lacking anti-HBV activity, highlighting the roles of hepatitis B vaccination, waning immunity, and anti-HBc status in driving reactivation or new infection.

Hepatitis B virus (HBV) infection remains an important clinical issue in people with HIV-1 (PWH), affecting both long-term health outcomes and the selection of appropriate antiretroviral therapy (ART), particularly when using regimens lacking anti-HBV activity, such as cabotegravir plus rilpivirine (CAB + RPV). This concern is particularly relevant in settings where universal HBV vaccination has not been fully implemented and where a substantial proportion of adults remain susceptible to HBV infection [1, 2].

In Japan, routine infant HBV vaccination was only introduced in 2016, later than in several neighboring Asian countries such as Taiwan, South Korea, and China [3–5]. Although these countries previously had a higher HBV prevalence than Japan, widespread vaccination programs have led to a marked decline in new HBV infections, making HBV a less prominent public health issue. In contrast, in Japan, new HBV infections—particularly those associated with sexual transmission in adulthood—continue to be reported. This is especially concerning among men who have sex with men (MSM), a key population at risk for HIV acquisition, in whom the risk of HBV infection also remains substantial. A cohort study of HIV-negative MSM has reported a high HBV incidence of approximately 3.8 per 100 person-years (PY) [6].

Among PWH, the cumulative prevalence of HBV exposure prior to ART initiation has been reported to be as high as 60% [7]. However, after the introduction of ART, the incidence of newly acquired HBV infection has been considered relatively low. This is largely attributable to the inclusion of agents with anti-HBV activity, such as tenofovir (TFV), lamivudine (3TC), and emtricitabine (FTC), in many ART regimens, which effectively function as daily pre-exposure prophylaxis (PrEP) against HBV [8].

However, the landscape of ART is rapidly evolving. With the introduction of regimens such as long-acting CAB + RPV [9] and the anticipated expansion in the use of agents such as lenacapavir (LEN) and islatravir (ISL), there is a growing shift toward ART regimens that lack anti-HBV activity [10–12]. As a result, PWH receiving these regimens may no longer be protected against HBV acquisition or reactivation. This issue is particularly important in regions like Japan, where universal vaccination coverage remains incomplete and HBV prevalence is still relatively high.

The objective of this study was to evaluate the incidence of newly acquired HBV infection and the clinically relevant risk of HBV reactivation among PWH receiving ART regimens without anti-HBV activity in Japan over the past two decades. By clarifying these risks in a setting without widespread universal vaccination, this study aims to inform optimal ART selection and HBV prevention strategies in PWH.

## MATERIALS AND METHODS

### Design and Study Population

This study was designed as a single-center, retrospective cohort study conducted at IMSUT Hospital, The Institute of Medical Science, The University of Tokyo. We included PWH who received care at our institution between January 2005 and March 2026 and who were treated with ART regimens that did not contain TFV, 3TC, or FTC, ie, regimens without anti-HBV activity.

Baseline data were collected from electronic medical records and included demographic and clinical characteristics such as HIV exposure category (men who have sex with men [MSM], heterosexual contact, or hemophilia), ethnicity, age at initiation of anti-HBV activity-free ART, CD4 and CD8 cell counts, and history of syphilis infection as assessed by Treponema pallidum hemagglutination assay (TPHA). In addition, HBV serological profiles at baseline, including hepatitis B surface antigen (HBsAg), anti-hepatitis B surface antibody (anti-HBs), and anti-hepatitis B core antibody (anti-HBc), were obtained. Baseline HBV DNA, HBeAg, and anti-HBe results were not used for HBV serological classification. We also collected information on detectable HIV viremia during the observation period. Detectable HIV viremia was defined as either two consecutive HIV RNA measurements ≥200 copies/mL or a single measurement ≥1000 copies/mL. The frequency of HBV serologic testing was calculated as the number of HBV serologic assessments per person-year during the observation period. An HBV serologic assessment was defined as a single blood sampling event with measurement of any HBV marker (HBsAg, anti-HBs, or anti-HBc); multiple markers measured on the same date were counted as one assessment.

Ethics approval was granted by the ethics board of the Institute of Medical Science, University of Tokyo (2022-48-1128). This study was reported in line with the Strengthening the Reporting of Observational Studies in Epidemiology (STROBE) guidelines.

### Definition of HBV Infection and Reactivation

Baseline HBV serological profiles were classified into five groups: (1) HBV-naïve individuals (anti-HBc negative, anti-HBs negative, and HBsAg negative), (2) HBV-naïve with vaccine-induced immunity (anti-HBc negative, anti-HBs positive, and HBsAg negative), (3) individuals with resolved HBV infection and anti-HBs positivity (anti-HBs positive, HBsAg negative, and anti-HBc positive, or anti-HBc negative with documented prior HBsAg positivity, detectable HBV DNA, or previous anti-HBc positivity), (4) isolated anti-HBc positivity (anti-HBc positive, anti-HBs negative, and HBsAg negative), and (5) chronic HBV infection (anti-HBc positive, anti-HBs negative, and HBsAg positive). These individuals were included because the study focused on withdrawal of anti-HBV-active ART rather than withdrawal of all HBV-directed therapy. Vaccination history was collected and defined as documented completion of a full 3-dose hepatitis B vaccination series in the medical records.

Incident HBV infection was defined among HBV-naïve individuals as the occurrence of acute hepatitis B, seroconversion to anti-HBc positivity, or development of anti-HBs positivity not attributable to vaccination. Acute hepatitis B was defined as clinically apparent hepatitis with elevated liver enzymes and evidence of newly acquired HBV infection.

Confirmed HBV reactivation was defined based on the 2025 European Association for the Study of the Liver (EASL) Clinical Practice Guidelines [13]. In HBsAg-positive, anti-HBc–positive individuals, it was defined as a ≥ 1 log10 increase in HBV DNA from baseline or HBV DNA ≥100 IU/mL after prior undetectable levels. In HBsAg-negative, anti-HBc–positive individuals, reactivation was defined by either HBsAg reappearance or detectable HBV DNA, with or without clinical hepatitis.

In addition, among individuals with isolated anti-HBc positivity who had maintained HBsAg negativity for more than 10 years—consistent with a functional cure—an increase in anti-HBs titers to ≥100 mIU/mL was classified as suspected HBV reactivation [13–15]. Although seroconversion to anti-HBs positivity may occur after ART initiation in isolated anti-HBc cases, in individuals with long-term stable functional cure, such a marked increase in anti-HBs is unlikely to represent a continuum of primary acute infection and was therefore interpreted as immune reactivation following previously suppressed HBV reactivation [16–18].

### Incidence of HBV Reactivation or Incident HBV Infection

The observation period was defined as the duration between the first and last blood sampling dates while the participant was receiving an ART regimen without anti-HBV activity. For participants who were already receiving such regimens at the start of the study period (January 2005), observation began at the date of the first available blood sampling after January 2005. Patients were excluded if their HBV serological profile prior to initiation of the relevant ART regimen was unavailable.

This was a retrospective study, and HBV serology and HBV DNA testing were not performed at predefined intervals but were obtained as part of routine clinical care. Testing was performed when clinically indicated (eg, elevated liver enzymes or suspected hepatitis) and was also obtained periodically at the discretion of the treating physician, particularly in individuals receiving ART regimens without anti-HBV activity. HBV DNA testing was not used as a routine screening test. Accordingly, the number and timing of HBV serologic tests varied across participants, reflecting real-world clinical practice.

For Kaplan–Meier analyses, the proportion of individuals remaining free from HBV reactivation or incident HBV infection was compared between anti-HBs–positive and anti-HBs–negative groups. The anti-HBs–positive group included individuals with vaccine-induced immunity and those with resolved HBV infection with anti-HBs positivity, whereas the anti-HBs–negative group included HBV-naïve individuals without anti-HBs, individuals with isolated anti-HBc positivity, and those with chronic HBV infection receiving entecavir. A sensitivity analysis excluding individuals with chronic HBV infection receiving entecavir was also performed. Among individuals with prior HBV exposure, an additional analysis was performed comparing confirmed and suspected HBV reactivation–free survival between individuals with resolved HBV infection with anti-HBs positivity and those with isolated anti-HBc positivity.

### Statistical Analysis

Demographic and clinical characteristics are presented as medians with interquartile ranges (IQR) for continuous variables and as frequencies and percentages for categorical variables.

Incidence rates were calculated based on person-time, without censoring participants after the occurrence of an event during the observation period. This approach was adopted because individuals, including those with anti-HBs positivity, may remain at risk for subsequent HBV reactivation. Incidence rates and corresponding 95% confidence intervals (CIs) were estimated assuming a Poisson distribution, using R version 4.5.1 (R Foundation for Statistical Computing, Vienna, Austria).

In Kaplan–Meier analyses, differences between groups were evaluated using the log-rank test. These analyses were conducted using Prism 10 (GraphPad Software, San Diego, CA, USA).

All statistical tests were two-sided, and statistical significance was defined as *P* < .05.

## RESULTS

### Study Population

A total of 166 PWH receiving antiretroviral regimens without anti-HBV activity were included in this study. Baseline characteristics are summarized in Table 1. The median age was 47 years (IQR, 39–54), and the median follow-up duration was 41.9 months (IQR, 27.7–44.9). A history of syphilis was documented in 92 individuals (56.1%). Detectable HIV viremia was observed in 7 individuals (4.2%). The median frequency of HBV serologic testing was 0.93 (IQR, 0.32–2.5) tests per person-year.

**Table 1.** Baseline and Demographic Characteristics

Total, n		166
Age		47 [39–54]
Gender, n (%)	Male	157 (94.6)
	Female^a^	9 (5.42)
Race, n (%)	Asians	158 (95.2)
	Southeast Asians	3 (1.81)
	Caucasians	3 (1.81)
	Latinos	2 (1.20)
HIV exposure category, n (%)	MSM	143 (86.1)
	Heterosexual contact	21 (12.7)
	Hemophilia (blood product exposure)	2 (1.20)
Detectable HIV viremia^b^, n (%)	…	7 (4.21)
History of syphilis^c^, n (%)	…	92 (56.1)
CD4 cell count, cells/μL	…	559 [426–707]
CD8 cell count, cells/μL	…	683 [554–908]
CD4/CD8 ratio	…	0.848 [0.530–1.11]
ART without anti-HBV activity^d^, n (%)	CAB + RPV	102 (61.5)
	DTG + boosted DRV	10 (3.61)
	DTG + RPV	6 (6.02)
	DTG + DOR	4 (2.41)
	Other NRTI-sparing regimens	19 (11.4)
	NRTI-containing regimens	25 (15.1)
Pre-switch ART with anti-HBV activity (any of tenofovir, FTC, or 3TC)^e^, n (%)	TDF/TAF + FTC/3TC	85 (51.2)
	3TC-containing regimen without tenofovir	57 (34.3)
	TDF-containing regimen without FTC/3TC	4 (2.41)
	No anti-HBV-active agents at study entry	20 (12.0)

Data are presented as n or median [IQR]. MSM, men who have sex with men; ART, antiretroviral therapy; CAB, cabotegravir; RPV, rilpivirine; DTG, dolutegravir; boosted DRV, darunavir/cobicistat or darunavir + ritonavir; DOR, doravirine; NRTI, nucleoside reverse transcriptase inhibitor; TDF, tenofovir disoproxil fumarate; TAF, tenofovir alafenamide; 3TC, lamivudine; FTC, emtricitabine.

^a^One participant was a transgender woman.

^b^≥2 consecutive HIV RNA ≥200 copies/mL or a single ≥1000 copies/mL.

^c^Based on TPHA positivity prior to baseline; 2 individuals had no prior TPHA testing.

^d^At baseline; some participants switched to other regimens without anti-HBV activity during follow-up.

^e^Pre-switch ART regimens reflect the regimen immediately prior to the switch; some participants may have a history of exposure to multiple prior ART regimens.

The distribution of baseline HBV serological patterns is shown in Table 2. A substantial proportion of individuals were HBV-naïve without anti-HBs, indicating a large susceptible population. In contrast, prior HBV infection was also common. Among individuals with chronic HBV infection, entecavir was used as HBV-directed therapy, which may have contributed to the prevention of HBV reactivation. All nine individuals with isolated anti-HBc had no history of HBV vaccination.

**Table 2.** Baseline HBV Serological Patterns Among Individuals Switching to Regimens Without Anti-HBV Activity

Total, n	…	166
HBV-naïve without anti-HBs^a^		57 (34.3)
—Vaccinated after switch		23
HBV-naïve with vaccine-induced immunity^b^		20 (12.0)
—Vaccinated before switch		4
Resolved HBV infection with anti-HBs positivity^c^		78 (47.0)
Isolated anti-HBc positivity^d^		9 (5.42)
Chronic HBV infection (HBsAg-positive)^e^		2 (1.20)

Anti-HBs, antibody to hepatitis B surface antigen; anti-HBc, antibody to hepatitis B core antigen; HBsAg, hepatitis B surface antigen.

^a^Anti-HBc negative, anti-HBs negative, and HBsAg negative.

^b^Vaccine-induced immunity was defined as anti-HBc negative, anti-HBs positive, and HBsAg negative, with documented prior HBV vaccination history. One individual had received a single booster dose immediately prior to switching after completing a prior 3-dose vaccination series, in the setting of waning anti-HBs titers, while the remaining three had completed a full 3-dose vaccination series.

^c^Anti-HBs positive, HBsAg negative, and anti-HBc positive, or anti-HBc negative with documented prior HBsAg positivity, detectable HBV DNA, or previous anti-HBc positivity.

^d^Anti-HBc positive, anti-HBs negative, and HBsAg negative.

^e^Anti-HBc positive, anti-HBs negative, and HBsAg positive. These two individuals were receiving entecavir.

### Incidence of HBV Reactivation and Incident Infection

During a total follow-up of 716 PY, five HBV-related events were observed. All events occurred in individuals who were anti-HBs negative at baseline. Of these, two were classified as incident HBV infections, and three were classified as HBV reactivation events. Among the HBV reactivation events, one was confirmed HBV reactivation, while the remaining two were suspected HBV reactivation based on changes in HBV serological markers. The overall incidence rate was 0.70 per 100 PY (95% confidence interval [CI], .23–1.63). Among anti-HBs-negative individuals (68 individuals, 391 PY), the incidence rate was 1.28 per 100 PY (95% CI, .41–2.98).

Among HBV-naïve individuals (with or without vaccine-induced immunity), two incident HBV infections occurred, with an incidence rate of 0.60 per 100 PY (95% CI, .07–2.17; PY = 334). Among HBV-naïve individuals lacking vaccine-induced immunity, the incidence rate was 0.76 per 100 PY (95% CI, .09–2.74; PY = 264).

Among individuals with evidence of HBV exposure (resolved HBV infection with anti-HBs positivity, isolated anti-HBc positivity, or chronic HBV infection on entecavir), the incidence rate of confirmed and suspected HBV reactivation was 0.78 per 100 PY (95% CI, .16–2.29; PY = 383). When individuals with chronic HBV infection receiving entecavir were excluded, the incidence rate of confirmed and suspected HBV reactivation among those with prior HBV exposure was 0.82 per 100 PY (95% CI, .17–2.39; PY = 367).

**Figure 1.**
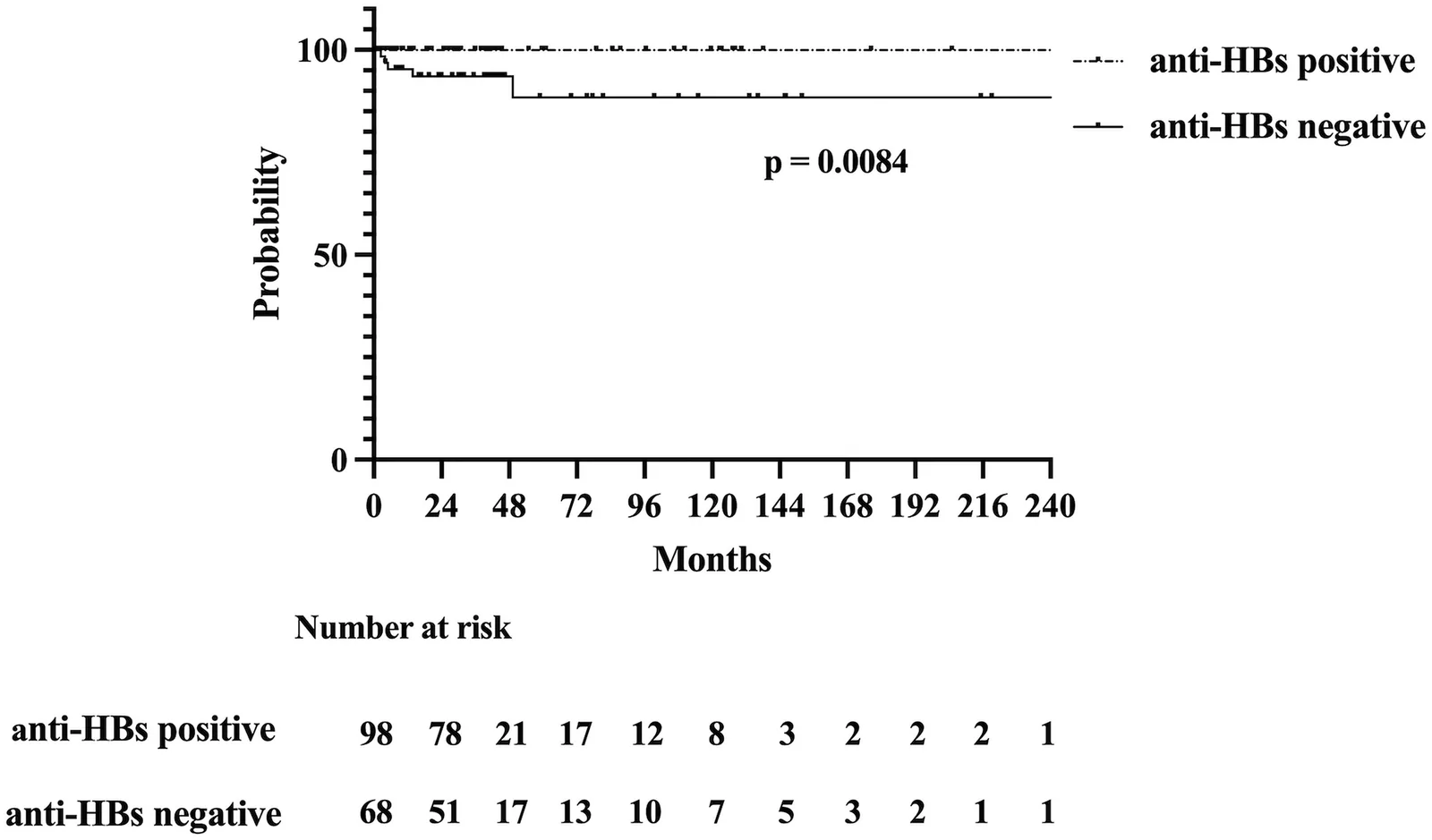
Kaplan–Meier analysis of HBV reactivation– or incident infection–free survival according to anti-HBs status. HBV event-free survival was compared between anti-HBs–positive and anti-HBs–negative groups. The anti-HBs–positive group included HBV-naïve individuals with vaccine-induced immunity and those with resolved HBV infection with anti-HBs positivity. The anti-HBs–negative group included HBV-naïve individuals without anti-HBs, individuals with isolated anti-HBc positivity, and those with chronic HBV infection. *P* values were calculated using the log-rank test.

Kaplan–Meier analysis demonstrated that the proportion of individuals remaining free from HBV reactivation or incident infection was significantly lower in anti-HBs–negative individuals than in anti-HBs–positive individuals (log-rank test, *P* = .0084) (Figure 1). A sensitivity analysis excluding individuals with chronic HBV infection receiving entecavir yielded similar results (log-rank test, *P* = .0072), with anti-HBs–negative individuals showing a significantly lower proportion remaining free from HBV events than anti-HBs–positive individuals. Among individuals with prior HBV exposure, the proportion remaining free from confirmed and suspected HBV reactivation was significantly lower in individuals with isolated anti-HBc positivity than in those with resolved HBV infection with anti-HBs positivity (log-rank test, *P* < .0001) (Supplementary Figure 1).

### Characteristics of HBV Events

Among the five cases of HBV reactivation or incident infection summarized in Table 3, two cases showed seroconversion to both anti-HBc and anti-HBs. In one of these cases, HBV vaccination was initiated after switching to CAB + RPV, and the infection was considered to have occurred prior to the development of protective immunity (Table 3, case 5). In both cases, no elevation of liver transaminases was observed and HBsAg remained negative throughout follow-up.

**Table 3.** HBV Reactivation and Incident Infection After Switching to Anti-HBV–Inactive Regimens

Case No.	Age	HBV Serological Profile	Pre Switch Regimen	Post-switch Regimen and Time To Event (months)	CD4 Count (at Switch), Cell/μL	Transaminase Elevation/Hepatitis Symptom	Diagnostic Evidence
1	60s	HBV-naïve without anti-HBs	TDF + LPVr + RAL	LPV/r + RAL, 49.2	290	None	Seroconversion of anti-HBs (15.1 mIU/mL) and anti-HBc (11.7 S/CO) without vaccination
2*	60s	Isolated anti-HBc	B/F/TAF	CAB + RPV, 13.8	531	None	Anti-HBs seroconversion; long-standing isolated anti-HBc (>20 y); anti-HBs increased to 317 mIU/mL after switch
3^a^	50s	Isolated anti-HBc^b^	B/F/TAF	CAB + RPV, 5.06	459	Yes (ALT elevation only)	Long-standing isolated anti-HBc (>10 y); HBV reactivation followed by chronic infection
4	60s	Isolated anti-HBc^b^	DTG/3TC	CAB + RPV, 2.43	1210	None	Anti-HBs seroconversion; Long-standing isolated anti-HBc (>10 y); anti-HBs increased to 1625 mIU/mL after switch
5	30s	HBV-naïve without anti-HBs	DTG/3TC	CAB + RPV, 3.91	874	None	Seroconversion of anti-HBs (818 mIU/mL) and anti-HBc (5.5 S/C) after the second dose of a three-dose HBV vaccination series

All cases occurred in men who have sex with men (MSM).

^a^Previously reported.

^b^Anti-HBs positivity was documented and subsequently lost, resulting in isolated anti-HBc positivity.

Abbreviations: anti-HBs, antibody to hepatitis B surface antigen; anti-HBc, antibody to hepatitis B core antigen; TDF, tenofovir disoproxil fumarate; LPV/r, lopinavir/ritonavir; RAL, raltegravir; B/F/TAF, bictegravir/emtricitabine/tenofovir alafenamide; CAB, cabotegravir; RPV, rilpivirine; DTG, dolutegravir; 3TC, lamivudine.

Two other cases had a history of anti-HBs positivity that subsequently waned, resulting in long-standing isolated anti-HBc patterns lasting for more than 20 years and more than 10 years, respectively (the former previously reported [15]). In both cases, marked increases in anti-HBs titers (317 mIU/mL and 1625 mIU/mL, respectively) were observed within several months after switching to CAB + RPV, as shown in Table 3, cases 2 and 4, suggesting an anamnestic immune response following withdrawal of HBV-active antiretroviral agents. According to the study definition, these cases were classified as suspected HBV reactivation. Neither case was associated with elevated liver transaminases, and no increase in HBsAg was documented following the serologic changes.

The remaining case, with isolated anti-HBc positivity, developed chronic HBV infection 5.1 months after the switch, as shown in Table 3, case 3. He developed HBsAg positivity, detectable HBV DNA, and HBeAg positivity accompanied by liver enzyme elevation. Prior to the switch, he had been receiving bictegravir/emtricitabine/tenofovir alafenamide (B/F/TAF), and following the diagnosis of HBV reactivation, the regimen was changed back to B/F/TAF. Detailed clinical, virological, and laboratory findings, including quantitative serologic results, HBV DNA levels, treatment course, and genomic analyses, have been described previously (previously reported [19]).

## DISCUSSION

In Japan, a representative country where universal HBV vaccination had not been historically implemented, previous studies have shown that incident HBV infection is rare among PWH receiving ART containing tenofovir disoproxil fumarate (TDF) or related agents [8]. In addition, cohort studies of MSM without HIV have demonstrated that HIV-PrEP exerts a preventive effect against HBV infection [6]. Because most ART regimens for PWH include agents with anti-HBV activity, such as TFV, 3TC, or FTC, PWH have effectively been under continuous HBV-PrEP. As a result, the intrinsic risk of HBV infection or reactivation in this population has likely been underestimated. In a prospective cohort study, Mizushima et al. reported an incidence rate of 3.8 per 100 PY among anti-HBs–negative MSM [6]. In our study, MSM-PWH without effective HBV-PrEP appeared to have a comparable level of risk. Although the incidence observed in our MSM-PWH subgroup limited to anti-HBs–negative individuals was lower than that reported in the MSM cohort, direct comparison is challenging due to differences in the definition and duration of the observation period, as well as differences in study populations, with our cohort also including non-MSM individuals.

We observed two cases with an isolated anti-HBc serological pattern who showed a marked and abrupt increase in anti-HBs titers. Isolated anti-HBc may represent a transitional phase of seroconversion, and anti-HBs can become detectable after ART initiation even if initially negative [20]. This phenomenon may be explained not only by immune reconstitution following ART but also by a reduction in HBsAg levels due to anti-HBV-active agents such as TFV, which may have previously masked anti-HBs detection [19]. However, in our two cases, long-term exposure to TFV or 3TC was followed by discontinuation of these agents, after which anti-HBs seroconversion occurred. This pattern is unlikely to be explained by a gradual decline in HBsAg levels and instead suggests a robust antibody response against HBsAg. Although it is difficult to distinguish between reactivation and incident infection based solely on serological status, the short observation interval and the absence of reported high-risk sexual exposure suggest that HBV reactivation is the more plausible explanation. In addition, long-term HBsAg negativity is generally considered indicative of functional cure [16]. In the present study, many individuals with isolated anti-HBc had a history of anti-HBs positivity, and none had received HBV vaccination. Given that HBV vaccination is generally recommended for individuals with isolated anti-HBc, vaccination may have the potential to prevent HBV reactivation in this population [21].

In Western cohorts, HBV reactivation among individuals with isolated anti-HBc positivity has been reported but appears to be infrequent [14, 22, 23]. For example, Denyer et al. reported a 1.02% rate of HBV reactivation among individuals with no prior HBsAg positivity and no prior anti-HBs positivity following switching from HBV-active to non–HBV-active antiretroviral therapy [22]. In contrast, our findings suggest that, in the setting of ART with anti-HBV activity, some individuals with isolated anti-HBc may have had underlying chronic HBV infection that was virologically suppressed and therefore masked. This may explain the occurrence of HBV-related events after discontinuation of anti-HBV-active agents in our cohort. Taken together, our findings suggest that in PWH receiving ART without anti-HBV activity, the risk of both HBV infection and reactivation becomes clinically apparent, particularly among those lacking anti-HBs and those with an isolated anti-HBc serological pattern, in settings where the impact of universal HBV vaccination remains limited.

In a Spanish cohort of 741 men with HIV, no cases of HBV reactivation were observed among individuals with isolated anti-HBc after switching to CAB + RPV [14]. In this cohort of 741 men with HIV, 22% had resolved HBV infection, 3% had isolated anti-HBc, 12% were HBV-naïve, and 61% had vaccine-induced immunity [14]. No cases of incident HBV infection or reactivation were observed after switching to CAB + RPV [14]. Compared with Japan, the proportion of individuals with vaccine-induced immunity was substantially higher, while the proportion of susceptible individuals was lower. In addition, the lower prevalence of chronic HBV infection in Spain may also have contributed to the absence of events.

Data from Spain, where HBV vaccination was initiated in adolescents in the 1990s and followed by a marked reduction in HBV prevalence and incidence, support the notion that vaccination during adolescence may lead to more rapid establishment of herd immunity [24]. In Japan, routine infant HBV vaccination was introduced in 2016; however, catch-up vaccination targeting adolescents could further enhance population-level protection and reduce the risk of HBV transmission.

This study has several limitations. First, it is a single-center retrospective cohort study. Although the cohort included a clinically relevant population at increased risk of HBV-related events, the small number of observed events limited statistical power and precluded robust multivariable analyses. Therefore, the incidence estimates should be interpreted with caution. Second, as a retrospective study, laboratory testing was not performed uniformly across all participants, which may have led to longer observation intervals and potential underestimation of incidence rates. This may partly explain why our observed incidence was lower than that reported in prospective studies. Nevertheless, our cohort includes PWH with diverse backgrounds in Japan, supporting the generalizability of our findings to real-world clinical settings. Finally, although we evaluated ART regimens without anti-HBV activity over a period spanning approximately two decades, approximately 60% of participants were receiving CAB + RPV, which became available in 2022, resulting in a bias toward more recent treatment patterns. However, this may also enhance the clinical relevance of our findings by reflecting current and future trends in HIV treatment.

The development of long-acting and non–HBV-active ART regimens, including CAB + RPV as well as emerging options such as DOR/ISL and bictegravir/lenacapavir (BIC/LEN), is expected to further reduce the use of reverse transcriptase inhibitors with anti-HBV activity [10, 11]. Although no incident infections were observed among anti-HBs–positive individuals in this study, waning vaccine-induced immunity is well recognized. Notably, Mizushima et al. reported a substantial incidence of HBV infection even among anti-HBs–positive MSM in Japan [6]. Therefore, in settings with a relatively high prevalence of HBV, careful evaluation of the impact of discontinuing the HBV-PrEP–like effect conferred by ART, along with appropriate preventive strategies, is warranted.

In conclusion, among MSM-PWH, HBV infection or reactivation was uncommon during ART containing anti-HBV agents such as TFV, 3TC, or FTC. After withdrawal of these agents, HBV-related events were observed exclusively among individuals without anti-HBs, suggesting increased vulnerability in this population, particularly in settings where the impact of universal HBV vaccination remains limited. Individuals with isolated anti-HBc may also warrant careful monitoring after discontinuation of anti-HBV-active ART. Further studies with larger cohorts are needed to better characterize HBV outcomes, including incident infection with seroconversion, progression to chronic infection, and reactivation and subsequent resolution, across different baseline HBV serological profiles.

## Supplementary Material

ofag533_Supplementary_Data

## Supplementary Data

Supplementary materials are available at *Open Forum Infectious Diseases* online. Consisting of data provided by the authors to benefit the reader, the posted materials are not copyedited and are the sole responsibility of the authors, so questions or comments should be addressed to the corresponding author.
