# Supplementary material for: Clinically Relevant Risk of Hepatitis B Virus Infection and Reactivation in People With HIV After Withdrawal of Anti-HBV–Active Antiretroviral Therapy in Japan
Source: Open Forum Infect Dis. 2026 Aug 20;13(9):ofag533. doi: 10.1093/ofid/ofag533 (PMC13532089; doi:10.1093/ofid/ofag533)
Supplement: Supplementary file 1 [file ofag533_supplementary_data.pdf]

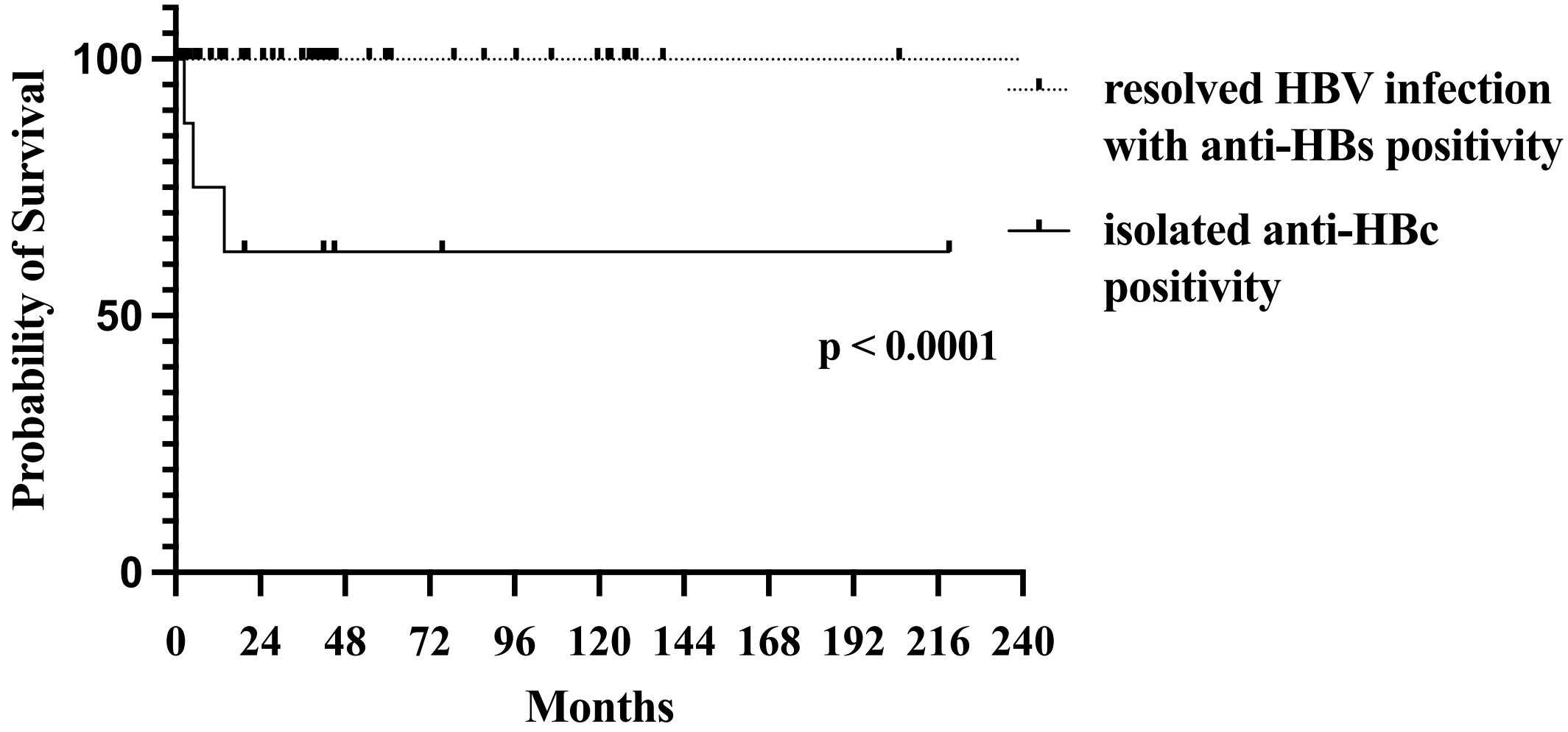

Number at risk

	0	24	48	72	96	120	144	168	192	216	240
**resolved HBV infection with anti-HBs positivity**	78	61	16	13	9	7	3	2	2	1	1
**isolated anti-HBc positivity**	9	3	3	2	1	1	1	1	1	1	0

Supplementary Figure 1. Kaplan–Meier analysis of confirmed and suspected HBV reactivation–free survival according to prior HBV exposure status.

Among individuals with prior HBV exposure, HBV reactivation–free survival was compared between those with resolved HBV infection with anti-HBs positivity and those with isolated anti-HBc positivity. Individuals with isolated anti-HBc positivity showed a significantly lower proportion remaining free from confirmed and suspected HBV reactivation compared with those with resolved HBV infection with anti-HBs positivity. P values were calculated using the log-rank test.
